# Inflammatory Markers of the Systemic Capillary Leak Syndrome (Clarkson Disease)

**DOI:** 10.4172/2155-9899.1000213

**Published:** 2014-04-30

**Authors:** Zhihui Xie, Eunice Chan, Yuzhi Yin, Chandra C. Ghosh, Laura Wisch, Celeste Nelson, Michael Young, Samir M. Parikh, Kirk M. Druey

**Affiliations:** 1 Laboratory of Allergic Diseases, NIAID/NIH, Bethesda, MD, USA; 2 Department of Medicine, Division of Nephrology and Center for Vascular Biology Research, Beth Israel Deaconess Medical Center and Harvard Medical School, Boston, MA, USA; 3 Clinical Research Directorate/CMRP, Leidos Biomedical Research Inc., Frederick, National Laboratory for Cancer Research, Frederick, MD, USA

**Keywords:** Systemic capillary leak syndrome, Inflammation, Cytokines, CXCL10, Monocytes

## Abstract

**Objectives:**

The Systemic Capillary Leak Syndrome (SCLS) is a rare and potentially fatal disorder resembling systemic anaphylaxis that is characterized by transient episodes of hypotensive shock and peripheral edema. The pathogenesis of SCLS is unknown, and triggers for attacks are apparent only in a minority of patients. We introduce a clinical algorithm for the diagnosis of SCLS, and we investigated potential serum biomarkers of acute SCLS episodes.

**Methods:**

We analyzed serum cytokines in a cohort of 35 patients with an established diagnosis of SCLS and characterized the effects of SCLS sera on endothelial cell function. We investigated the cellular source(s) of CXCL10, a chemokine that was significantly elevated in both basal and acute SCLS sera, by flow cytometry.

**Results:**

Several cytokines were elevated in acute SCLS sera compared to baseline or sera from healthy controls, including CXCL10, CCL2, IL-1β, IL-6, IL-8, IL-12 and TNFα. The majority of acute sera failed to activate endothelial cells as assessed by surface adhesion marker expression. Monocytes appear to be the major source of serum CXCL10, and the percentage of CXLC10+ monocytes in response to IFNγ stimulation was increased in SCLS subjects compared to controls.

**Conclusions:**

The presence of proinflammatory cytokines in acute SCLS sera suggests that inflammation or infection may have a role in triggering episodes. The enhanced capacity of monocytes from SCLS patients to produce CXCL10 suggests a new therapeutic avenue for SCLS.

## Introduction

The Systemic Capillary Leak Syndrome (SCLS) is a life-threatening disorder that occurs sporadically, typically in middle-aged Caucasians [1,2]. SCLS is characterized by recurrent, reversible episodes of distributive shock and anasarca due to sudden and unexplained leakage of plasma into the tissues. SCLS resembles, and is often erroneously diagnosed as, more common disorders such as sepsis, angioedema, and systemic anaphylaxis. Although a monoclonal gammopathy of unknown significance (MGUS) is highly associated with SCLS [3,4], its pathophysiologic significance is unclear. Therefore, because no specific biomarkers or pathognomonic clinical sign(s) of SCLS exist currently, the triad of transient hypotension, hemoconcentration, and serum hypoalbuminemia in the absence of underlying causes is sufficient to establish the diagnosis [2,5,6]. Fewer than 250 cases have been reported since Clarkson described SCLS in 1960 [2]. There is no specific treatment for acute SCLS other than hemodynamic support with intravenous fluids and vasopressors. Drugs such as theophylline, β2-adrenergic agonists, and most recently, imatinib, have been used as prophylactic agents to promote vascular integrity in SCLS [6]. Based on anecdotal evidence accumulated over several years, it has become apparent that monthly intravenous immunoglobulin (IVIG, 2 g/kg) infusions have severely curtailed attacks in a majority of patients who receive this therapy indefinitely [6–8].

Most research findings on SCLS have been based on single case reports, or purely clinical observations [2,6]. Using samples from our (then) 23-patient cohort, we reported previously that sera from SCLS patients during acute disease intervals uniformly induced hyperpermeability of human microvascular endothelial cell (HMVEC) monolayers due to disruption of endothelial adherens junctions and actin stress fiber formation [4]. In contrast, sera from these patients obtained during remission had little to no impact on HMVEC barrier integrity and resembled sera from healthy controls. HMVEC hyperpermeability elicited by acute SCLS sera was inhibited by pretreatment of target endothelial cells with IVIG or by antibody neutralization of Angiopoietin-2 (Ang2), a known mediator of vascular permeability that was elevated in acute SCLS sera compared to sera from healthy donors [4].

Although the trigger(s) of acute SCLS episodes are unclear, a longitudinal case registry study of 28 SCLS patients found that about three quarters of subjects experienced flu-like symptoms prior to attack onset [6]. Analysis of our expanded cohort (35 subjects) revealed elevated levels of several proinflammatory cytokines in acute sera, particularly CXCL10. Monocytes from a subset of these patients demonstrated an increased capacity for CXCL10 production. These results suggest a potential role for CXCL10 in the initiation of acute SCLS.

## Materials and Methods

### Study subjects

Patients were diagnosed with SCLS and classified according to established criteria described previously [2,6]. Patients were seen at the Clinical Center of the National Institutes of Health. Written informed consent was obtained from each patient, and the study protocol (09-I-0184) conformed to the ethical guidelines of the 2008 Declaration of Helsinki as reflected in a priori approval from the Institutional Review Board of the National Institute of Allergy and Infectious Diseases of NIH. Age-, sex-, and race-matched serum samples and lymphapheresis samples were obtained from the NIH Blood Bank.

### Cells and reagents

Human umbilical vascular endothelial cells (HUVECs) were purchased from American Tissue Type Collection. Endothelial Growth Medium (EGM) 2 was obtained from Lonza. AlexaFluor 647-conjugated anti-ICAM1, phycoerythryin-conjugated anti-VCAM1, anti-CXCL10, phycoerythryin/Cy7-conjugated anti-CD14 antibodies and the protein transport inhibitor monensin were purchased from BioLegend. Phycoerythryin-Cy5-conjugated anti-E-selectin and APC-conjugated anti-CD19 antibodies were obtained from BD Biosciences. Qdot 605-conjugated anti-CD3 and Live/Dead fixable violet dead stain kit were from Life Technologies. Recombinant human TNFα and IFNγ, and CXCL10 Quantikine ELISA kit were purchased from R & D Systems.

### Cytokine measurements

Serum cytokines were measured using a Bio-plex Pro human cytokine 27-plex and a customized human cytokine 9-plex assay kit purchased from Bio-Rad according to the manufacturer’s instructions.

### Endothelial activation marker analysis

HUVECs were seeded in 6-well plate and incubated overnight at 37°C. Cells were incubated with test serum (10% vol/vol final concentration) in growth medium for 4 hours. The cells were then detached with CellStripper (Mediatech), washed once with PBS, and stained with the indicated antibodies for 30 minutes. Cells were then washed twice, fixed with 4% paraformaldehyde for 10 minutes, and analyzed by flow cytometry.

### CXCL10 measurement by ELISA

HUVECs were seeded in 96-well plate overnight at 37°C, and followed by incubation with test sera (10% vol/vol final concentration) in growth medium for 24 hours. Cell culture supernatants were collected and analyzed for CXCL10 by ELISA.

### PBMC isolation

Peripheral blood mononuclear cells (PBMCs) were isolated from lymphapheresis samples using lymphocyte separation medium (LSM, LP BioMedicals). Briefly, apheresis samples were diluted 1:1 in PBS, and 30 ml of diluted sample was overlaid on onto LSM (15 ml) in a 50 ml conical tube. Samples were centrifuged at 800 × g in a tabletop centrifuge at room temperature for 40 minutes with break off.

### Flow cytometric analysis of CXCL10 production

PBMCs were left untreated or stimulated with IFNγ (20 ng/ml) in the presence of monensin overnight in 37°C. The following day cells were stained with Live/Dead violet staining kit according to manufacturer’s instructions and fixed in 4% paraformaldehyde. Fixed cells were permeabilized with blocking buffer (0.1% saponin, 1 mM CaCl_2_, 1 mM MgSO_4_, 0.1% BSA, 10 mM HEPES, 5% milk, and 5% human Fc receptor inhibitor, in PBS) for 30 minutes followed by staining with antibodies in blocking buffer for additional 30 min. Stained cells were washed twice and analyzed using LSRII flow cytometer (BD BioSciences).

### Statistical analysis

Data were analyzed with the GraphPad Prism 6 software package. 1-way ANOVA or Kruskal-Wallis (for multiple groups) was used for grouped cytokine analyses, and unpaired parametric Student’s *t* test was used for flow analysis of CXCL10 production. *P* values < 0.05 were considered significant.

## Results

### Algorithm for the diagnosis of SCLS

**Figure 1:**
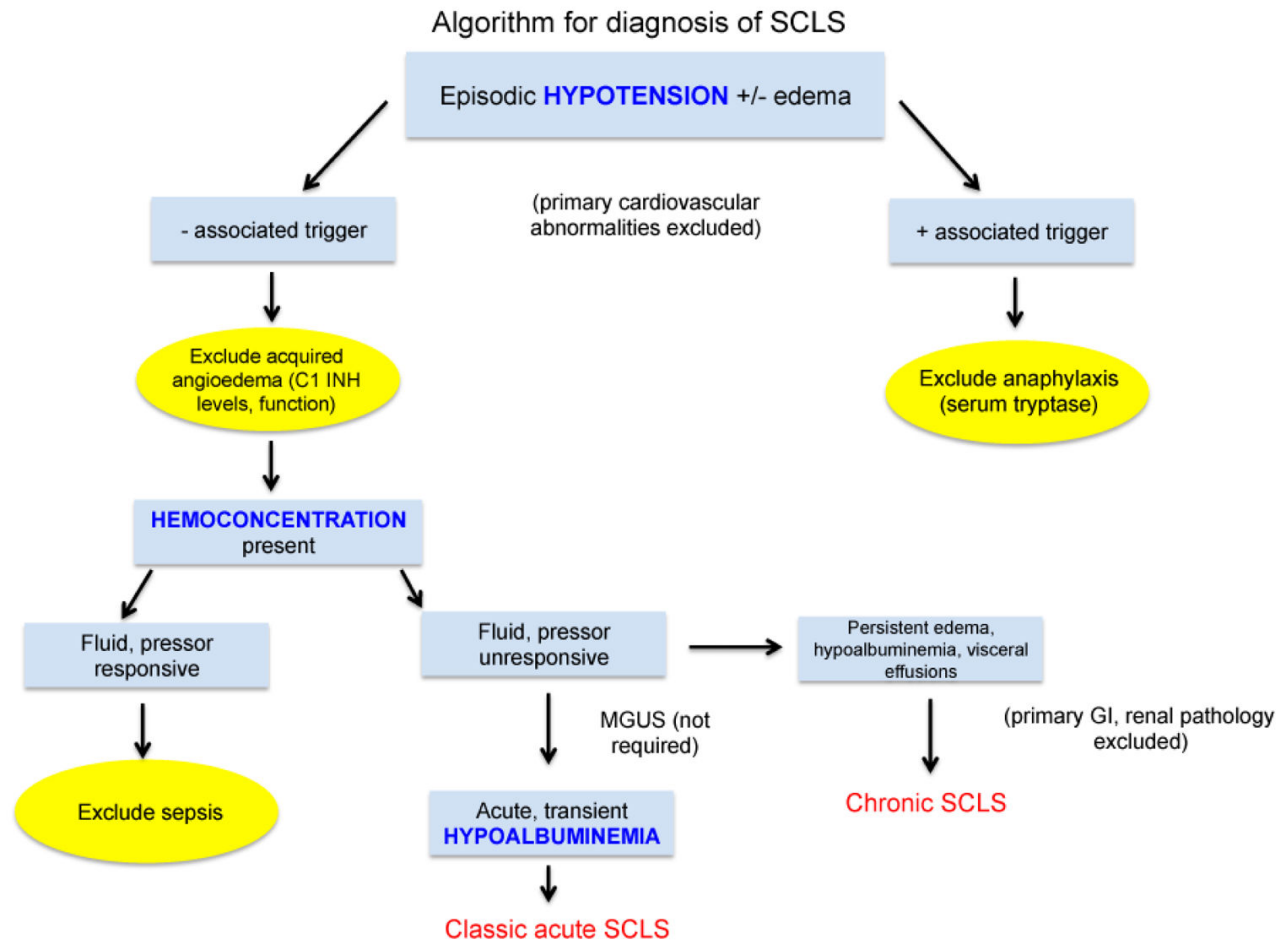
Clinical considerations in the prospective diagnosis of SCLS. After exclusion of primary cardiovascular and/or allergic causes, a diagnosis of SCLS should be entertained in patients with unexplained, transient hypotension and/or peripheral edema. Systemic anaphylaxis and hereditary and/or acquired angioedema can be excluded by measurement of serum tryptase during the acute episode and quantitative and functional assays for the complement component 1 esterase inhibitor (C1 INH). Although presumptive treatment for sepsis is prudent in the undiagnosed SCLS patient during the first severe episode, the hypotension and hemoconcentration of SCLS are typically refractory to intravenous fluid resuscitation, which exacerbates peripheral edema. Hypoalbuminemia due to protein extravasation is a hallmark of classic acute SCLS whereas low serum albumin levels and edema that does not resolve between episodes should prompt the diagnosis of “chronic” SCLS. MGUS is not universally present in SCLS and is therefore not required for the diagnosis.

SCLS should be considered in a patient with unexplained, transient hypotension and/or peripheral edema (Figure 1). If a temporally linked exposure (e.g. food, insect venom, drug) is suspected, elevated serum tryptase should exclude systemic anaphylaxis. Although our experience and that of others [6,9] suggest that acute triggers for SCLS attacks are often absent, viral-type upper respiratory and/or systemic symptoms may be present in many patients with SCLS prior to the onset of an episode. While catastrophic SCLS attacks are typically accompanied by massive edema of the face, trunk, and peripheral extremities, swelling resembling angioedema may be confined to certain areas (periorbital, back, and abdomen) in less severe episodes. Thus, complement factor 1 esterase inhibitor (C1 INH) levels and function should be evaluated in all patients with suspected SCLS to rule out hereditary or acquired angioedema.

A hallmark of severe acute SCLS episodes is hemoconcentration due to the loss of water and solutes into the extravascular space. Marked elevations in serum hemoglobin over the patient’s baseline, often greater than 20 g/dL, are common, occasionally leading to an erroneous diagnosis of polycythemia vera [10]. In contrast to cases of dehydration and sepsis, the hemoconcentration and hypotension of SCLS do not reverse immediately following administration of intravenous fluids and/or vasopressors. Central venous pressures remain low (typically <2 mm Hg) in the acute SCLS leak phase, and massive intravenous saline infusion often aggravates peripheral edema and can elicit compartment syndromes in the extremities.

Serum hypoproteinemia due to protein extravasation is universally present in acute SCLS, and albumin levels of <2 g/dL are common. Some patients may present with persistent, noncyclical, peripheral edema and hypoalbuminemia, and this subset may or may not experience acute hypotensive episodes. These patients are classified as having “chronic” SCLS and may also present with visceral (pleural, pericardial) effusions. Finally, it should be emphasized that although MGUS is present in 85–95% of SCLS cases, it is not required for the diagnosis.

### Characteristics of the SCLS study cohort

We evaluated 35 patients with a confirmed diagnosis of SCLS based on the criteria outlined above and exclusion of other primary causes of hypotension and/or edema (Table 1). The median age at the time of the SCLS diagnosis was 46 years (range 22 months to 66 years) although the vast majority of patients experienced clinical symptoms attributable to SCLS for several years prior to receiving the actual diagnosis. All but one patient was Caucasian, and 43% were female. Our cohort included three children and three adult patients with chronic edema. Most subjects experienced at least two episodes of SCLS per year, but the range varied widely, from once in five years to weekly. Overall, 86% had MGUS, and 27/29 M-proteins were of the IgG isotype. M-proteins were not detected in any of the children, whereas they were present in 91% of adults with SCLS. While most patients were treated with theophylline and terbutaline following diagnosis, 79% (23/29) experienced breakthrough episodes while on this regimen. Currently, 63% are treated with monthly infusions of IVIG (2 g/kg), and the majority of these patients (20/22) have been essentially episode-free for periods of up to eight years. One patient, a seven-year old child, is receiving subcutaneous IG because of IVIG intolerance. Two somewhat atypical patients (Pt. 3 and Pt. 31) experienced recurrent episodes of idiopathic hypotension, hemoconcentration, and hypoalbuminemia consistent with the diagnosis of SCLS, yet presented with unique symptomatology and did not respond at all to standard SCLS therapy, including IVIG. These patients also had M-protein isotypes unusual for SCLS (IgA or IgM, respectively).

### Serum cytokine analysis of SCLS

**Figure 2:**
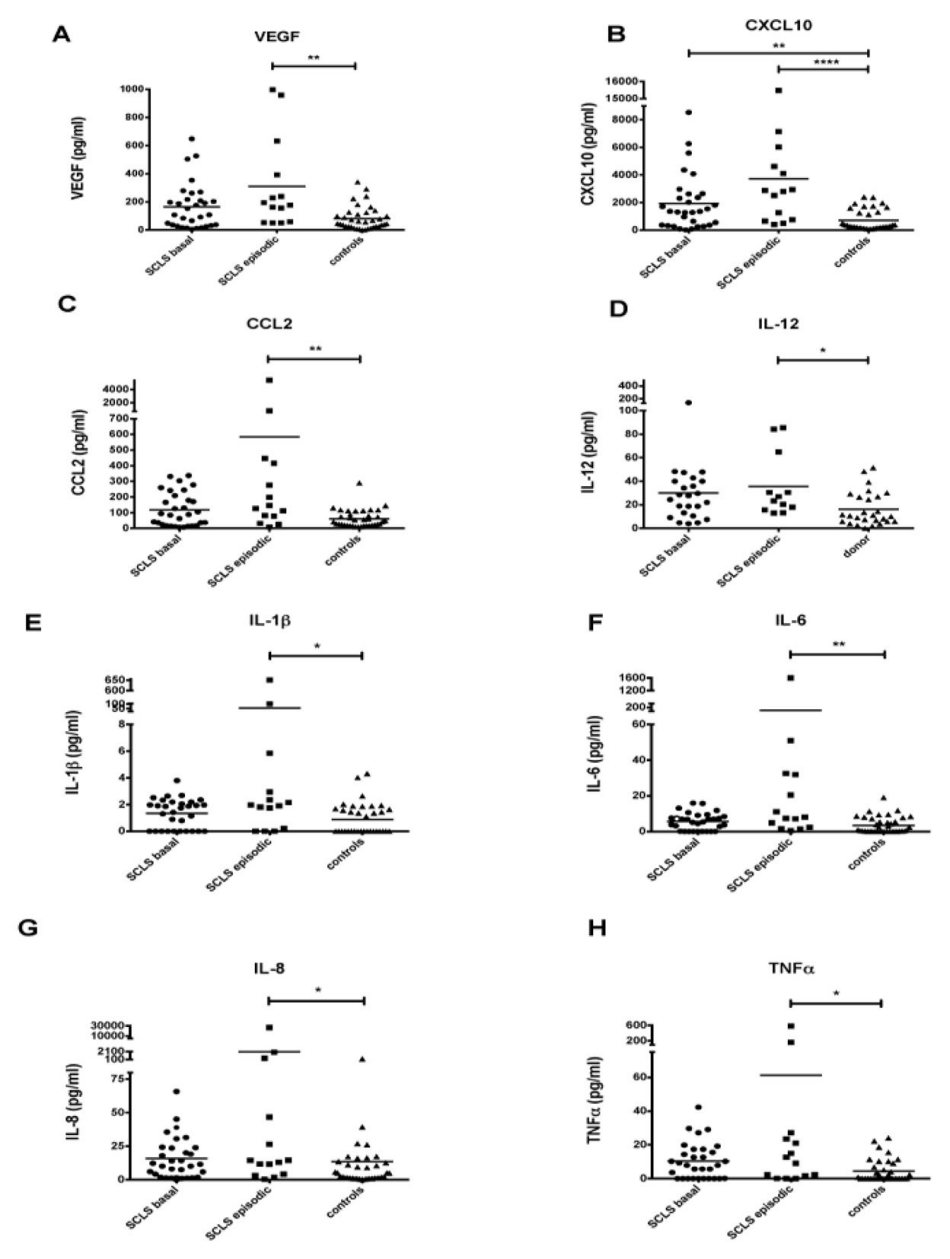
Cytokine profiles of SCLS sera. (A–H) Serum cytokines were measured using a Bio-Plex Pro human multiple cytokine assay kit: VEGF (A), CXCL10 (B), CCL2 (C), IL-12 (D), IL-1β (E), IL-6 (F), IL-8 (G) and TNFα (H) (*P < 0.05, **P < 0.01, ****P < 0.00001 Kruskal-Wallis).

Although nearly one quarter of subjects reported flu-like symptoms prior to the onset of a full-blown attack [4], whether this prodrome represents a concomitant infection or is a primary feature of SCLS is unknown. Our previous studies that include a cohort of 23 SCLS subjects demonstrated that some (VEGF, Ang 2), but not all (IL-2), soluble mediators of inflammation were increased in acute SCLS sera [4]. To assess the role of infection and/or inflammation in acute SCLS episodes and to discover new mediators that may contribute to disease onset and/or severity, we analyzed a panel of 27 serum cytokines in sera from 31 SCLS patients in remission, 14 SCLS patients with acute symptoms (samples were not available from all patients), and 37 healthy controls by multiplex bead assay. Ten cytokines (IL-2, IL-4, IL-5, IL-10, IL-13, IL-15, IL-17, G-CSF, GM-CSF, CCL3) were absent or present at very low levels in most sera tested, while levels of the cytokine RANTES were present at levels above the detectable assay range in all samples. Quantities of eight cytokines (IL-1RA, IL-7, IL-9, bFGF, PDGFb, CCL4, and CCL11) did not differ significantly among the groups or between individual SCLS subjects. Consistent with our previous findings, VEGF was increased in acute SCLS sera compared with sera from healthy controls (Figure 2A). We detected significantly increased levels of seven cytokines in acute SCLS sera relative to sera from healthy donors, specifically, CXCL10, CCL2, IL-12, IL-1β, IL-6, IL-8, and TNFα (Figures 2B–2H). Of all cytokines measured, only CXCL10 was significantly increased both in baseline and acute SCLS sera relative to controls (Figure 2B). These data suggested that TH1-associated inflammation or infection is associated with acute SCLS episodes.

### Activation of vascular endothelial cells by acute SCLS sera

**Figure 3:**
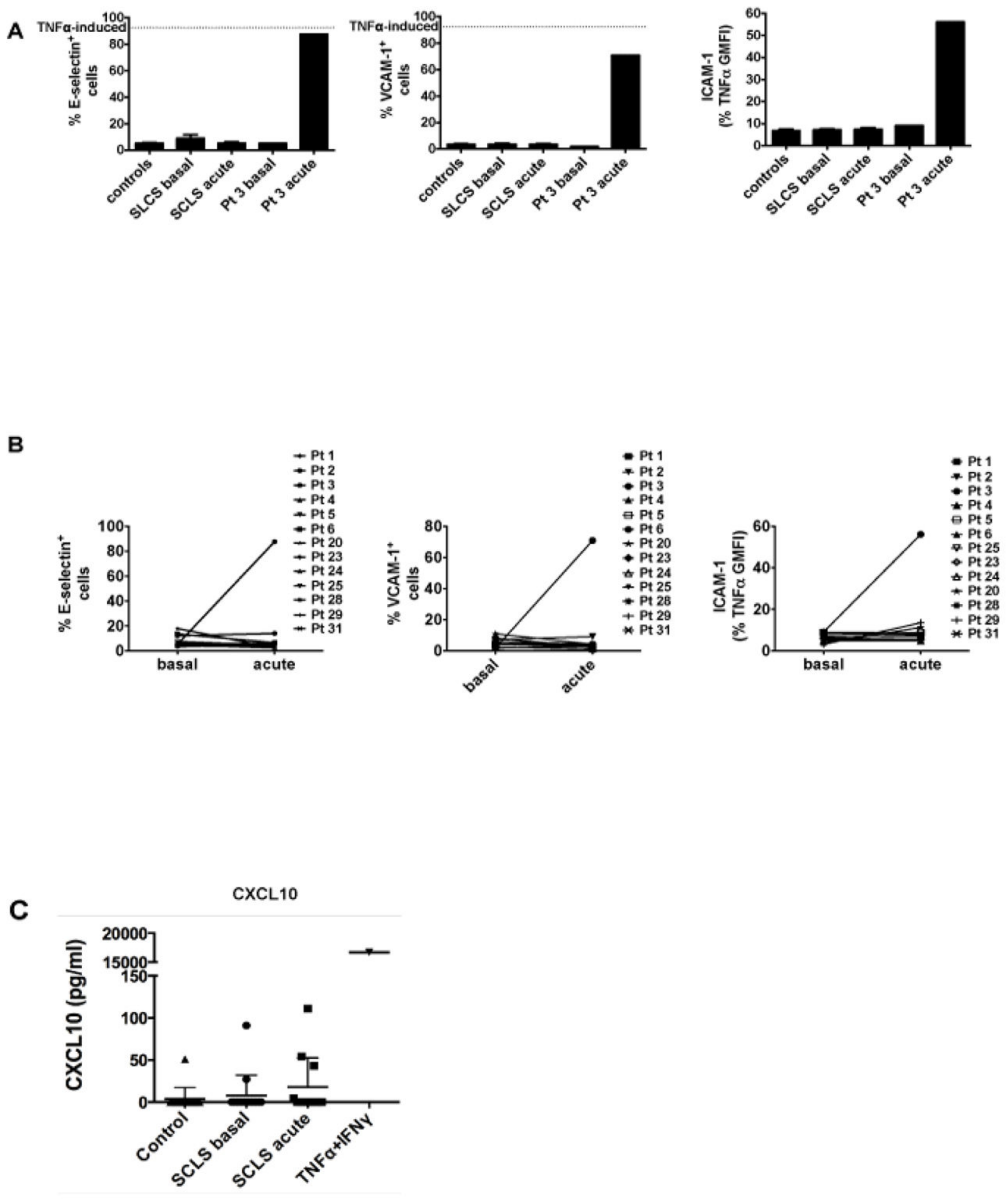
Endothelial surface activation marker expression induced by SCLS sera. HUVECs were incubated with 10% (vol/vol) test sera for 4 hours (A–B) or 24 hours (C) at 37°C. (A) Surface expression of E-selectin, VCAM-1 and ICAM-1 was evaluated by flow cytometry. The dotted lines represent adhesion molecule expression induced by TNFα (1.25 ng/ml). (B) Paired comparisons of E-selectin, VCAM-1 and ICAM-1 expression induced by basal and episodic sera from each individual subject. (C) CXCL10 in HUVEC culture supernatants was measured by ELISA.

Skin biopsies of SCLS patients have revealed the presence of perivascular leukocytic inflammation during acute episodes in some cases [11]. Proinflammatory cytokines including TNFα and IL-8 have been shown to increase adhesion molecule surface expression on endothelial cells, which in turn promotes leukocyte rolling, migration and extravasation [12]. To determine whether factors in SCLS sera could evoke endothelial barrier dysregulation by inducing leukocyte adhesion, we incubated HUVECs with acute and baseline sera from SCLS subjects or healthy donors and analyzed surface expression of E-selectin, VCAM-1 and ICAM-1 by flow cytometry. Unexpectedly, among the SCLS sera tested (20 baseline and 13 acute) only Pt. 3’s episodic serum induced upregulation of these adhesion molecules in HUVECs (Figure 3A–B). Surface expression of these markers induced by Pt. 3’s episodic sera was comparable to that evoked by TNFα (Figure 3A, dotted line) [13], a well-known endothelial activator that served as positive control for these experiments. The concentration of TNFα in Pt. 3’ episodic serum (top square dot in Figure 2G) is comparable to that used in control experiments, 20-fold higher than that found in most of other SCLS sera (Figure 2G). These results and the unresponsiveness of HUVECs to acute SCLS serum samples containing low levels of TNFα suggest that TNFα is probably the cytokine that mediated endothelial adhesion/activation marker upregulation in Pt. 3’s episodic sera. Since endothelial cells have the capacity to produce CXCL10 in response to inflammatory stimuli [14], we examined whether they produced CXCL10 following treatment with SCLS sera. We incubated HUVECs with acute or baseline sera from healthy controls or subjects with SCLS for 24 hours and measured CXCL10 in culture supernatants. TNFα plus IFNγ served as a positive control, as described in previous work [14,15]. None of the SCLS sera tested elicited CXCL10 secretion from HUVECs (Figure 3C). Taking together, these results suggested that in classic SCLS, endothelium is probably not the major source of CXCL10.

### Increased CXCL10-producing monocytes in SCLS subjects

**Figure 4:**
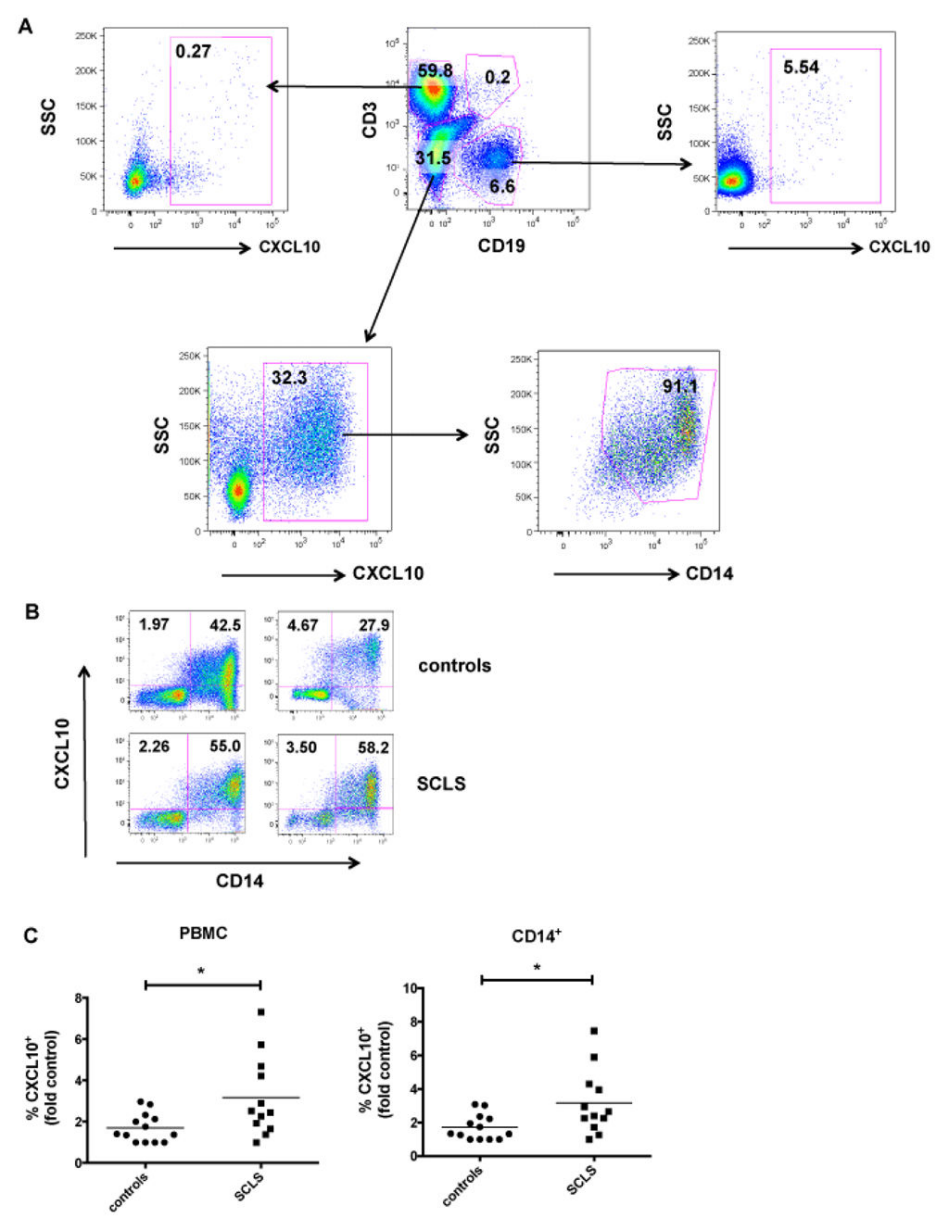
Abnormal IFNγ-induced CXCL10 production by PBMCs in SCLS. PBMCs were incubated with IFNγ and analyzed for CXCL10 production by flow cytometry. (A) Percentages of CXCL10+ cells among T lymphocytes (CD3+), B lymphocytes (CD19+), and monocyte (CD14+) populations from a representative healthy subject. (B–C) Representative flow plots (B) and analysis of CXCL10+ PBMCs and monocytes from SCLS subjects (n = 12) and healthy subjects (n = 14), presented as the fold control of the lowest value from a healthy donor for each individual experiment, set as ‘1’. *P = 0.02, unpaired t test.

CXCL10, which is induced by many proinflammatory factors such as IFNγ, is secreted by many cell types including monocytes, endothelial cells, and fibroblasts during inflammation or infections [16]. To determine the cellular source of CXCL10 in SCLS sera, we studied whether CXCL10 production by peripheral blood mononuclear cells differed in control subjects and those with SCLS. As expected, percentages of PBMCs producing CXCL10 constitutively were low (data not shown), but INFγ elicited dramatic increases in the number of CXCL10^+^ cells (Figure 4A). Whereas percentages of CXCL10^+^ T and B lymphocytes were low, about 30% of monocytes from healthy control subjects produced CXCL10 following IFNγ stimulation. Greater than 90% of all CXCL10-producing non-B non-T cells were monocytes (CD14^+^) (Figure 4A, lower right panel). Following IFNγ treatment, we detected a significantly higher number of CXCL10^+^ mononuclear cells and CXCL10^+^ monocytes in SCLS subjects compared to healthy controls (Figures 4B–4C). These results suggested that monocytes from subjects with SCLS have increased responsiveness to an inflammatory stimulus (IFNγ).

## Discussion

Among an extensive group of proinflammatory cytokines studied, we found that elevations in CCL2, IL-1β, IL-6, IL8, IL-12 and TNFα were associated with acute SCLS episodes, and CXCL10 levels were both constitutively and acutely increased in SCLS sera. CXCL10 appears to be derived principally from peripheral monocytes, and SCLS patients have significantly more circulating monocytes with the capacity to make CXCL10 than controls.

We demonstrated increased VEGF levels in acute SCLS sera, which corroborated our previous ELISA results in sera from nine SCLS subjects (also included in the current group) [4], indicating that VEGF is a potential mediator of endothelial barrier dysfunction in SCLS. Although symptoms associated with viral infections do not precede SCLS episodes in all cases, some patients report episodes triggered by infections. Accordingly, we found increased quantities of CCL2, IL-1β, IL-6, IL-8, IL-12 and CXCL10 in SCLS acute sera, suggesting that type 1 immune responses T_H_1 (e.g. chronic viral, bacterial, or protozoan infections), rather than T_H_2 allergic immunity, may contribute the SCLS phenotype in at least a fraction of patients.

Of all cytokines studied, CXCL10 was prominently elevated in both basal and acute SCLS sera relative to controls. CXCL10 has been shown to inhibit endothelial cell proliferation and induce apoptosis [17], a mechanism previously implicated in the pathogenesis of SCLS [18,19]. However, we were unable to demonstrate endothelial apoptosis induced by acute SCLS sera in our previous study [4]. In the current work, acute SCLS sera failed to activate endothelial cell adhesion marker expression (with one exception), and none of the sera elicited CXCL10 production by endothelial cells, suggesting that endothelial cells may not be a major source of CXCL10 in SCLS. Although IFNγ is a strong inducer of CXCL10, many T_H_1-associated cytokines are able to increase CXCL10 production by monocytes and macrophages. Because IFNγ levels were similar in SCLS and control sera, other factors including VEGF, TNFα, IL-β and IFNγ may act synergistically to elicit CXCL10 production by monocytes/macrophages in SCLS [20–22]. IFNγ-independent CXCL10 production may also occur [23]. For example, a single polynucleotide polymorphism (SNP) in the *Cxcl10* promoter (−135G>A) enhanced its transcriptional activity in tuberculosis [24]. Thus, mutations or polymorphisms in *Cxcl10* could also account for aberrant CXCL10 production in SCLS.

Because elevated serum levels of CXCL10 have been observed in many diseases including rheumatoid arthritis, systemic lupus erythematosus, systemic sclerosis [25], and HIV infection [16], it probably has no utility as a specific diagnostic marker for SCLS. However, it may be useful for diagnosis and/or prognosis when considered with previously identified permeability mediators VEGF and Ang2. CXCL10 is a potent chemoattractant for monocytes/macrophages and activated T cells and promotes leukocyte transmigration through endothelium into inflamed tissues. Further study of abnormal CXCL10 production and its cellular source(s) may provide insights into the initial permeability-inducing trigger in SCLS episodes as well as uncover new therapeutic strategies for this potentially fatal disorder.

## Figures and Tables

**Table 1:** Clinical profiles of SCLS subjects.

Pt no.	Age at Diagnosis*	Sex	Ethnicity	Attack Frequency	Current Treatment Regimen	MGUS
1	45	M	C	Every 4–6 weeks	IVIG	IgG kappa
2	49	F	C	Weekly/monthly	IVIG	IgG kappa
3	45	M	C	Every weekend	Infliximab	IgA kappa
4	55	M	C	> 2/yr	IVIG	IgG kappa
5	46	F	C	5–10 day intervals	N/A (deceased)	IgG lambda
6	42	M	C	3–4 year intervals	Theophylline	IgG kappa
7	32	M	C	3–5 year intervals	IVIG	IgG kappa
8	59	M	C	Single episode	None	IgG kappa
9	65	M	C	3–4/year	IVIG	IgG kappa
10	39	F	C	Chronic	T/T**	ND#
11	48	F	C	Infrequent	T/T	IgG lambda
12	48	M	C	weekly	IVIG	IgG kappa
13	50	F	C	> 2x/yr	IVIG	IgG kappa
14	51	M	C	> 2x/yr	IVIG	IgG
15	53	M	C	Weekly	IVIG	IgG lambda
16	40	F	C	Chronic	Theophylline	IgG kappa
17	43	F	C	3–4 year interval	None	IgG kappa
18	64	F	C	Chronic	Theophylline	IgG kappa
19	48	M	AA	3–4/yr	IVIG	IgG lambda
20	43	M	C	Bimonthly	IVIG	IgG kappa
21	65	M	C	Single episode	Theophylline	IgG kappa
22	43	M	C	< 2x/yr	IVIG	IgG kappa
23	36	F	C	< 2x/yr	IVIG	IgG kappa
24	66	M	C	< 2x/yr	IVIG	IgG lambda
25	40	F	C	Single episode	IVIG	IgG kappa
26	62	M	C	< 2x/yr	IVIG	IgG kappa
27	7	F	C	Chronic plus acute	Subcutaneous IG	ND#
28	48	M	C	< 2x/yr	IVIG	IgG lambda
29	61	M	C	3–4 year intervals	IVIG	IgG kappa
30	43	F	C	< 2x/yr	IVIG	IgG lambda
31	58	F	C	Chronic plus acute	None	IgM kappa
32	33	M	C	< 2x/yr	IVIG	ND#
33	37	F	C	Chronic plus acute	IVIG	ND#
34	22 mo.	F	C	Every 5–6 months		ND#
35	8	C	C	Weekly	IVIG	ND#

* Note: Many patients experienced episodes consistent with SCLS prior to formal diagnosis;

** : Theophylline plus terbutaline;

# : Not detected

## Abbreviations

SCLS: Systemic Capillary Leak Syndrome
HUVEC: Human Umbilical Vein Endothelial Cells
PBMC: Peripheral Blood Mononuclear Cells
ICAM-1: Intercellular Adhesion Molecule-1
VCAM-1: Vascular Cell Adhesion Molecule-1
IVIG: Intravenous Immunoglobulin
IFNγ: Interferon Gamma
TNFα: Tumor Necrosis Factor α
VEGF: Vascular Endothelial Growth Factors
Ang2: Angiopoietin 2
MGUS: Monoclonal Gammopathy of Unknown Significance
